# Erector Spinae Plane Blocks With Liposomal Bupivacaine for Pediatric Scoliosis Surgery

**DOI:** 10.5435/JAAOSGlobal-D-21-00272

**Published:** 2022-01-21

**Authors:** Casey Stondell, Rolando Roberto

**Affiliations:** From the Shriners Hospitals for Children Northern California (Dr. Stondell, and Dr. Roberto), and the University of California Davis Medical Center (Dr. Roberto), Sacramento, CA.

## Abstract

Pain management in patients with adolescent idiopathic scoliosis (AIS) undergoing posterior spinal fusion can be challenging. Various analgesic techniques are currently used, including enhanced recovery after surgery principles, spinal opioids or continuous epidural infusion, intravenous methadone, or surgical site infiltration of local anesthetic. Another recently developed technique, ultrasound-guided erector spinae plane blockade (ESPB), has been used successfully in spine surgery and may offer advantages because of its ease of placement, excellent safety profile, and opioid sparing qualities. Liposomal bupivacaine is a long-acting local anesthetic that was recently approved for infiltration and fascial plane blocks in pediatric patients of ages 6 years and older. This medication may prove to be beneficial when administered through ESPB in patients with AIS undergoing posterior spinal fusion because it can provide prolonged analgesia after a single injection. Here, we present a case report of two such patients, and we compare outcomes with a retrospective cohort of 13 patients with AIS who received IV methadone instead of ESPB. ESPB patients seemed to have less opioid use and shorter length of stay but higher pain scores, although the sample size is too small for meaningful statistical analysis. Future prospective trials are needed to see if differences in outcomes truly exist.

Pain management for adolescent patients with idiopathic scoliosis (AIS) undergoing posterior spinal fusion (PSF) can be challenging. Inadequately controlled pain and adverse effects from opioids continue to be common barriers to discharge.^1,2^

Neuraxial analgesia through spinal opioids or continuous epidural infusion can provide excellent pain control, but these techniques have drawbacks. They can lead to rare but serious complications, such as infection, hematoma, or nerve injury, often cause severe pruritus, nausea, vomiting, and urinary retention, and may cause hypotension in the postoperative period.^3-5^

Another analgesic strategy that has gained in popularity in the past few years in this patient population is intravenous methadone. Using methadone avoids the risks associated with neuraxial techniques, and a single dose can provide analgesia for up to 72 hours after complex spine surgery and was not associated with increased adverse events when compared with intravenous narcotics.^6^ However, a large initial dose is required to achieve long-lasting effects, potentially leading to adverse effects common to all opioids, such as sedation, urinary retention, and constipation.^7^

Ultrasound-guided erector spinae plane blockade (ESPB) was first developed by Forero in 2016 as an easy, safe way of managing thoracic neuropathic pain.^8^ Since that time, this block has gained in popularity and has been used to effectively provide analgesia for a variety of surgeries, including spine surgery.^9-12^ ESPB may also have a better safety profile when compared with neuraxial analgesia, perhaps because of direct visualization of the needle under ultrasonography guidance and the ease of placement, although this has not been decisively proven.^13-15^ One disadvantage of this block is the short duration of action from a single injection. Continuous ESPB catheters have been described, although placement before incision would not be possible in PSF, and nerve block catheters are prone to complications and failure.^16,17^

Liposomal bupivacaine (LB) is a long-acting local anesthetic that has recently been approved for infiltration and fascial plane blocks in pediatric patients of ages 6 years and older. Pharmacokinetic studies in this population have shown that, when administered at the recommended maximum dose of 4 mg/kg, plasma levels of bupivacaine are well below toxic levels, confirming the safety of this medication in children.^18^ After being injected, the liposomes are slowly metabolized over multiple days, leading to a controlled, sustained release of bupivacaine. Surgical site infiltration of LB has been effective in controlling pain in pediatric patients undergoing spine surgery, but its use in ESPB in this population has not been evaluated to our knowledge.^19^ In an attempt to achieve long-lasting analgesia while minimizing perioperative opioids, we modified our existing enhanced recovery after spine surgery (ERAS) protocol to include preoperative four-point ESPB with LB instead of IV methadone (Table 1). Here, we describe the courses of two such patients. To put the outcomes into context, we also reviewed the charts of 13 consecutive patients with AIS undergoing PSF who received IV methadone during the 6 months before converting to ESPB (Table 2).^20^

**Table 1:** Standardized Perioperative Enhanced Recovery After Surgery Protocol

Preoperative	Intraoperative	Postoperative
Daily MiraLax (polyethylene glycol) starting 3 days before surgery	Ketamine 1 mg/kg (max 100 mg) with induction then 5 μg·kg^−1^·min^−1^ infusion throughout surgery	Acetaminophen 15 mg/kg IV/PO Q6 hr
Carbohydrate beverage (clear liquid) 2 bottles the evening before surgery 2 bottles the morning of surgery (finished at least 2 hours before start time)	Dexamethasone 0.2 mg/kg up to 10 mg	Ketorolac 0.5 mg/kg IV Q 6 hr (first dose 12 hr after celecoxib administration. Transition to oral ibuprofen on POD 3)
Scopolamine patch	TIVA with propofol and remifentanil infusions	Oxycodone 0.1 mg/kg PO Q6 hr for 24 hr, then switch to PRN only
Oral acetaminophen 15 mg/kg	Tranexamic acid 50 mg/kg load then 10 mg·kg^−1^·hr^−1^ infusion	Gabapentin 5 mg/kg (max dose 300 mg) PO QHS (stop POD 4)
Oral celecoxib <50 kg: 100 mg >50 kg: 200 mg	Baseline MEPs and SSEPs followed by preincision ESPB with LB LB 4 mg/kg mixed with bupivacaine 0.25% (up to 2 mg/kg) Volume expanded with saline or lactated ringer if needed to reach total volume of 60 mL Divide expected incision into thirds, bilateral injections at upper and lower thirds, 15 mL each	PRN PO oxycodone, IV hydromorphone, IV/PO diazepam Daily physical therapy with a goal of walking on POD 1 or 2
Type and screen for curves less than or equal to 70°, otherwise type and cross 1 unit	Additional opioid per anesthesiologist discretion	Scheduled PO colace (docusate) and senna, PO MiraLax on POD 2 if no bowel movement, and bisacodyl suppository on POD 3 if no bowel movement

ESPB = erector spinae plane blockade, LB = liposomal bupivacaine, MEPs = motor-evoked potentials, POD = postoperative day, SSEPs = somatosensory-evoked potentials, TIVA = total intravenous anesthesia, PO = by mouth, PRN = as needed, QHS = at bedtime

**Table 2:** Characteristics and Outcomes: ESPB with LB Versus IV Methadone

	Surgery Duration (min)	No. of Levels Fused	Total Inpatient OMEs mg/kg Through POD 3	Average VAS on POD 1	Average VAS on POD 2	Average VAS on POD 3	LOS (min)
Case 1	297	10	1.67	4.4	2.6	6.4	3630
Case 2	242	9	1.85	4.7	5.4	5.1	3830
Methadone group (N = 13, means)	283	9	3.5	3.4	4	3.4	5490

Mean IV methadone dose 0.27 mg/kg.

OMEs = oral morphine equivalents, POD = postoperative day, LOS = length of stay, VAS = visual analog pain scale, ESPB = erector spinae plane blockade, LB = liposomal bupivacaine

A conservative 1:2 ratio was used to convert intravenous methadone to intravenous morphine.^20^

## Case Reports

**Figure 1:**
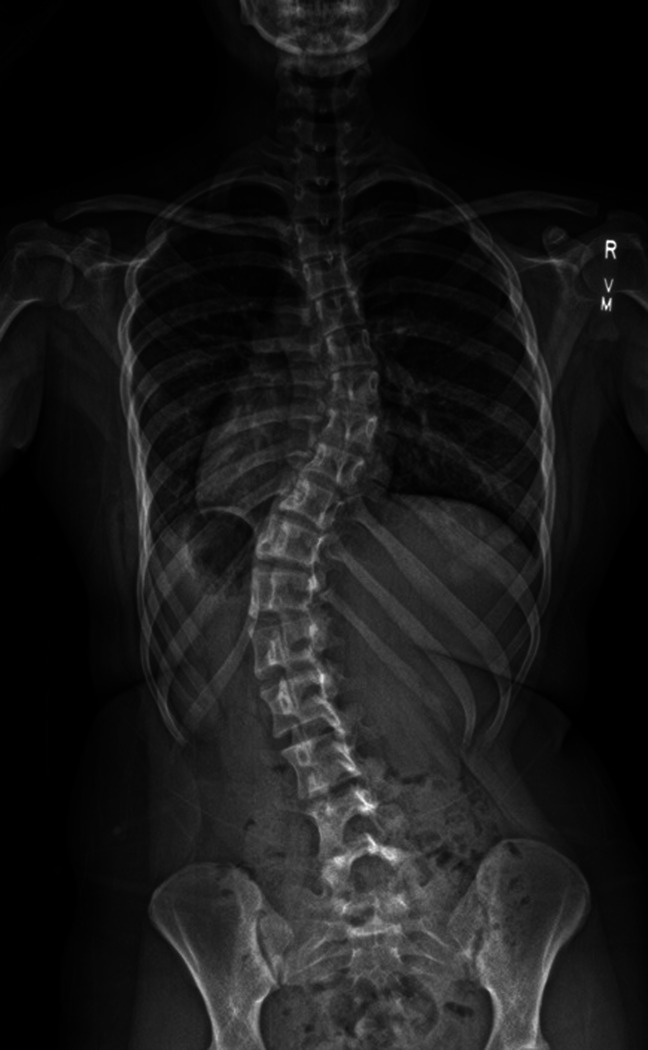
Preoperative radiograph of a patient who received erector spinae plane block with liposomal bupivacaine.

**Figure 2:**
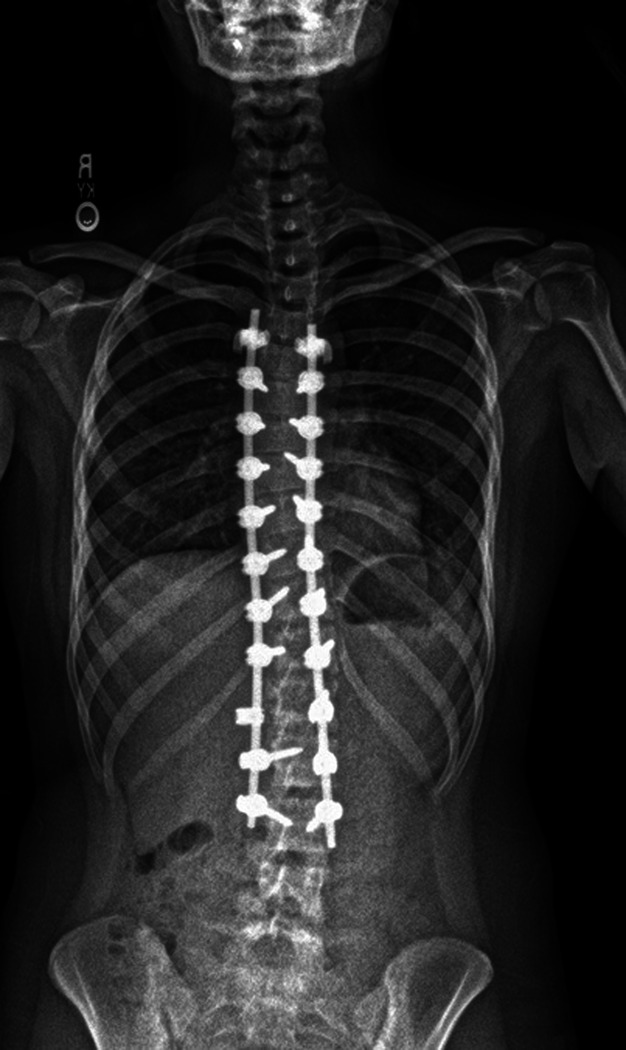
Postoperative radiograph of a patient who received erector spinae plane block with liposomal bupivacaine.

Case 1: A 16-year-old, 60 kg female adolescent with a history notable only for AIS presented for PSF from T5 to L3. On preoperative imaging, she was noted to have thoracic dextroconvex curvature measuring 39° and thoracolumbar levoconvex curvature measuring 44° (Figure 1). She received perioperative multimodal management per our standardized ERAS protocol (Table 1). Before incision, normal baseline somatosensory-evoked potentials (SSEPs) and motor-evoked potentials (MEPs) were obtained. Before incision, four-point bilevel ESPBs were done with an admixture of LB and bupivacaine hydrochloride. Bupivacaine hydrochloride was added to provide immediate analgesia because there is a delay in peak efficacy of LB. Cranial-femoral traction was initiated; this is standard practice at our institution, even for nonsevere curves. Lumbar pedicle screws were placed uneventfully, and SSEPs and MEPs remained stable until thoracic pedicle screws were placed, at which point a 90% reduction in MEPs was noted only on the left side. The mean arterial pressure was increased to 85 using a phenylephrine infusion, and cranial-femoral traction was reduced. MEPs and SSEPs quickly came back to baseline and remained normal throughout the rest of the surgery. Postoperative radiographs showed excellent correction of the spinal deformity (Figure 2). The patient was admitted to the intensive care unit (ICU) overnight for blood pressure management with a goal mean arterial pressure of 85 to 95 to optimize spinal cord perfusion. Her pain was well-controlled (Table 2). She was transferred to the floor on postoperative day (POD) 1. She was discharged on POD 3. At her 4-week follow-up appointment, the patient's pain was well-controlled with acetaminophen and ibuprofen.

Case 2: A 16-year-old, 48 kg female adolescent with a history of AIS, anxiety, and depression presented for PSF from T5 to L2. Preoperative imaging showed thoracic dextroscoliosis measuring 46° and mild lumbar scoliosis of 17°. She received standard perioperative multimodal management (Table 1). Normal MEPs and SSEPs were obtained before ESPB with LB/bupivacaine hydrochloride; they remained at baseline throughout the duration of the surgery. Cranial-femoral traction was applied; her surgery was uneventful, and she was admitted to the floor postoperatively where her pain was fairly well-controlled (Table 2). She was discharged on POD 3. At her 4-week follow-up visit, she was only taking acetaminophen and ibuprofen; she had not used any opioids for 3 weeks.

## Erector Spinae Plane Block Technique

**Figure 3:**
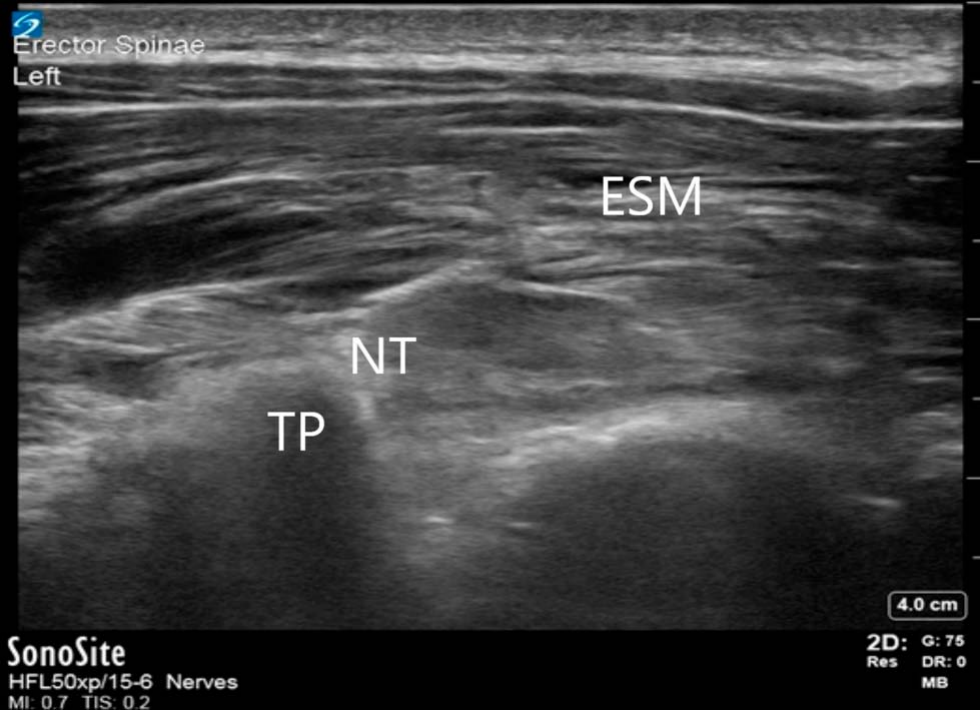
Ultrasound image of an erector spinae plane block. ESM = erector spinae muscle group, NT = needle tip, TP = transverse process

**Figure 4:**
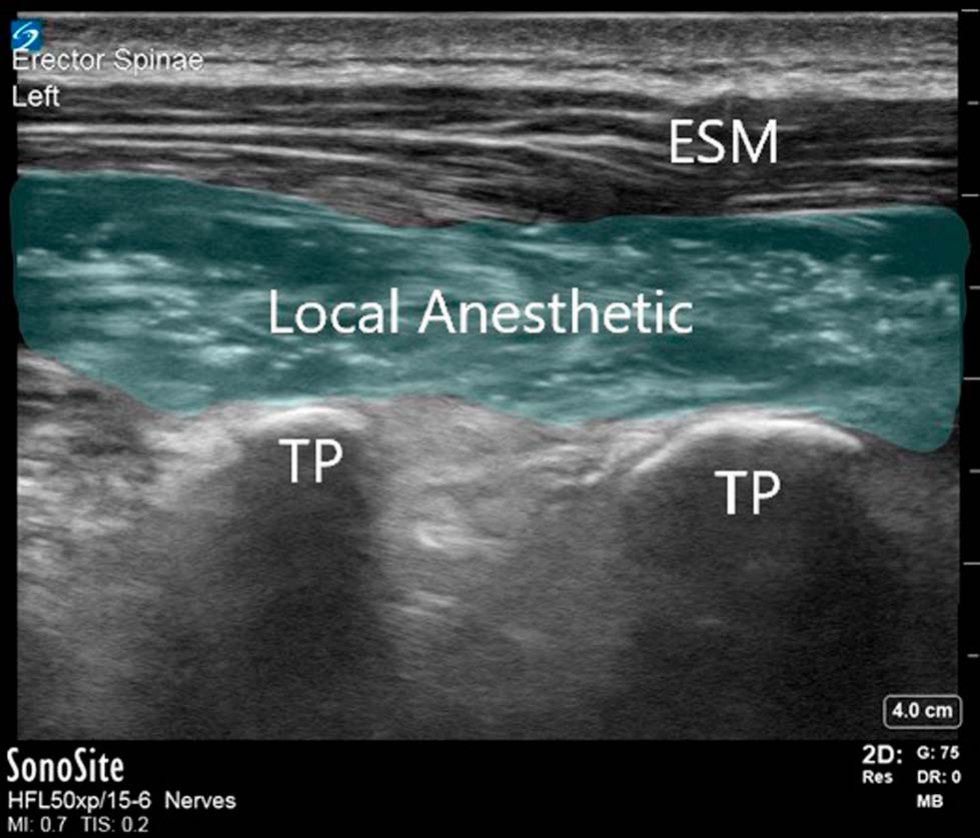
Ultrasound image of local anesthetic spread during erector spinae plane block. ESM = erector spinae muscle group, TP = transverse process

After turning prone onto the operating room table, baseline MEPs and SSEPs are obtained. The surgeon then marks the length of the anticipated incision, and this is divided into thirds. A timeout is done confirming the patient's identity and planned regional anesthesia technique. The back is prepped and draped. A linear ultrasonography probe is then used to identify bilateral transverse processes at the upper and lower thirds of the incision (Figure 3). To minimize the risk of contaminating the injection sites (standing on the patient's right side, holding the ultrasonography probe in the left hand, and directing the needle with the right hand), we prefer to perform the blocks in the following order: lower right, lower left, upper right, and upper left. A needle is advanced in plane toward the transverse process until the bone is encountered; the needle is then withdrawn slightly. Saline is injected to confirm adequate spread in the erector spinae muscle group plane, and then the local anesthetic mixture is injected (Figure 4). The recommended dose of LB for pediatric patients is 4 mg/kg with a maximum dose of 266 mg. We mix the LB with up to 2 mg/kg of bupivacaine hydrochloride to achieve immediate analgesia in addition to long-lasting analgesia. We also add epinephrine 5 mcg/mL as a marker for inadvertent vascular injection. Fifteen milliliters is injected at each of the four sites; if needed, saline or lactated ringers can be added to the mixture for volume expansion.

## Discussion

Currently, no consensus is observed regarding the optimal analgesic strategy for patients with AIS undergoing PSF, yet providing adequate postoperative pain control while minimizing opioids, is an imperative part of ERAS. Achieving these goals allows for early mobilization and decreases medication adverse effects, including nausea, vomiting, and constipation, which can all contribute to delayed discharge.^21^ Decreasing perioperative opioid consumption by combining safe, effective regional anesthesia techniques with multimodal analgesia should be prioritized in this patient population.

The mechanism of action of ESPB is still controversial because some studies show that local anesthetic may spread to the epidural and/or paravertebral space while other studies have shown only fascial spread.^22^ Regardless of the degree of spread, there is general agreement that ESPB does block spinal dorsal rami and does result in somatic and visceral analgesia.^23^ Some have voiced concern about the potential for ESPB to interfere with MEPs and/or SSEPs during spine surgery, yet this concern has not been validated in the literature.^10,11,24^ In the first case report we described, the patient did experience a transient substantial decrease in MEPs, but this was unilateral, it was well after ESPB was done, and it occurred in conjunction with pedicle screw placement during heavy traction. It was also quickly reversed after increasing the blood pressure and decreasing the traction. No issues were observed with MEPs or SSEPs in the second case.

LB is approved for infiltration and fascial plane blocks in pediatric patients of ages 6 years and older at a dose of 4 mg/kg up to 266 mg. Peak analgesia after injection is delayed because of its slow release, which is why we mix it with bupivacaine hydrochloride (and saline or lactated ringers if more volume is needed). Studies have shown LB to be effective at providing long-lasting analgesia in pediatric patients undergoing spine surgery but, to the best of our knowledge, this is the first description of its use in ESPB for patients with AIS undergoing PSF.^19^ Compared with our prior standard analgesic technique of IV methadone, the two patients presented here who received ESPB with LB had shorter LOS and less opioid consumption but seemed to have higher average pain scores. Because the sample size of the ESPB patients is so small, it is not appropriate to evaluate these outcomes for statistical significance, but it does show that this technique may be a reasonable alternative to other currently used analgesic strategies. Prospective, randomized trials will be necessary to elucidate whether there truly are differences in outcomes.

## References

[1] Chan P Skaggs D Sanders A : Pain is the greatest preoperative concern for patients and parents prior to posterior spinal fusion for adolescent idiopathic scoliosis. Spine 2017;42:1245-1250. 10.1097/BRS.0000000000002147 28263228

[2] Muhly WT Sankar WN Ryan K : Rapid recovery pathway after spinal fusion for idiopathic scoliosis. Pediatrics 2016;137:e20151568. 27009035 10.1542/peds.2015-1568

[3] Gramigni E Bracco D Carli F: Epidural analgesia and postoperative orthostatic haemodynamic changes: Observational study. Eur J Anaesthesiol 2013;30:398-404. 23435278 10.1097/EJA.0b013e32835b162c

[4] Wood CE Goresky GV Klassen KA Kuwahara B Neil SG: Complications of continuous epidural infusions for postoperative analgesia in children. Can J Anaesth 1994;41:613-620. 8087910 10.1007/BF03010002

[5] Christie IW McCabe S: Major complications of epidural analgesia after surgery: Results of a six-year survey. Anaesthesia 2007;62:335-341. 17381568 10.1111/j.1365-2044.2007.04992.x

[6] Gottschalk A Durieux ME Nemergut EC: Intraoperative methadone improves postoperative pain control in patients undergoing complex spine surgery. Anesth Analg 2011;112:218-223. 20418538 10.1213/ANE.0b013e3181d8a095

[7] Stemland C Witte J Colquhoun D : The pharmacokinetics of methadone in adolescents undergoing posterior spinal fusion. Pediatr Anesth 2012;23:51-57. 10.1111/pan.12021 22978825

[8] Forero M Adhikary SD Lopez H Tsui C Chin KJ: The erector spinae plane block: A novel analgesic technique in thoracic neuropathic pain. Reg Anesth Pain Med 2016;41:621-627. 27501016 10.1097/AAP.0000000000000451

[9] Kendall MC Alves L Traill LL De Oliveira GS: The effect of ultrasound-guided erector spinae plane block on postsurgical pain: A meta-analysis of randomized controlled trials. BMC Anesthesiol 2020;20:99-11. 32357842 10.1186/s12871-020-01016-8 PMC7195766

[10] Diwan SM Yamak Altinpulluk E Khurjekar K Nair A Dongre H Turan A: Bilateral erector spinae plane block for scoliosis surgery: Case series. Rev Esp Anestesiol Reanim 2020;67:153-158. 10.1016/j.redar.2019.11.012 32057483

[11] Melvin JP Schrot RJ Chu GM Chin KJ: Low thoracic erector spinae plane block for perioperative analgesia in lumbosacral spine surgery: A case series. Can J Anaesth 2018;65:1057-1065. 29704223 10.1007/s12630-018-1145-8

[12] Vergari A Frassanito L Muro M : Bilateral lumbar ultrasound-guided erector spinae plane block versus local anesthetic infiltration for perioperative analgesia in lumbar spine surgery: A randomized controlled trial. Research Square, preprint, version one. 2021. 10.21203/rs.3.rs-464372/v1. Accessed October 18, 2021. 35191639

[13] Tsui BCH Fonseca A Munshey F McFadyen G Caruso TJ: The erector spinae plane (ESP) block: A pooled review of 242 cases. J Clin Anesth 2019;53:29-34. 30292068 10.1016/j.jclinane.2018.09.036

[14] Tulgar S Selvi O Senturk O Serifsoy TE Thomas DT: Ultrasound-guided erector spinae plane block: Indications, complications, and effects on acute and chronic pain based on a single-center experience. Cureus 2019;11:e3815. 30868029 10.7759/cureus.3815 PMC6402723

[15] Chin KJ Dinsmore MJ Lewis S Chan V: Opioid-sparing multimodal analgesia with bilateral bi-level erector spinae plane blocks in scoliosis surgery: A case report of two patients. Eur Spine J 2020;29:138-144. 31482311 10.1007/s00586-019-06133-8

[16] Ahsan Z Carvalho B Yao J: Incidence and failure of continuous peripheral nerve catheters for postoperative analgesia in upper extremity surgery. J Hand Surg Am 2014;39:324-329. 24480691 10.1016/j.jhsa.2013.11.011

[17] Jeng CL Torrillo TM Rosenblatt MA: Complications of peripheral nerve blocks. Br J Anaesth 2010;105(suppl 1):i97-107. 21148659 10.1093/bja/aeq273

[18] Tirotta C J de Armendi A Horn N : A multicenter study to evaluate the pharmacokinetics and safety of liposomal bupivacaine for postsurgical analgesia in pediatric patients aged 6 to less than 17 years (PLAY). J Clin Anesth 2021;75:110503. 34534923 10.1016/j.jclinane.2021.110503

[19] Chughthai M Sultan A Hudson B : Liposomal bupivacaine is both safe and effective in controlling postoperative pain after spinal surgery in children. Clin Spine Surg 2020;33:E533-E538. 32324672 10.1097/BSD.0000000000000996

[20] Von Korff M Saunders K Ray T : Defacto long-term opioid therapy for non-cancer pain. Clin J Pain 2008;24:521-527. 18574361 10.1097/AJP.0b013e318169d03b PMC3286630

[21] Lee CS Merchant S Chidambaran V: Postoperative pain management in pediatric spinal fusion surgery for idiopathic scoliosis. Paediatr Drugs 2020;22:575-601. 33094437 10.1007/s40272-020-00423-1

[22] Chin KJ El-Boghdadly K: Mechanisms of action of the erector spinae plane (ESP) block: A narrative review. Can J Anaesth 2021;68:387-408. 33403545 10.1007/s12630-020-01875-2

[23] Chin KJ Malhas L Perlas A: The erector spinae plane block provides visceral abdominal analgesia in bariatric surgery: A report of 3 cases. Reg Anesth Pain Med 2017;42:372-376. 28272292 10.1097/AAP.0000000000000581

[24] Tsui BCH Esfahanian M Lin C Policy J Vorhies J: Moving toward patients being pain- and spasm-free after pediatric scoliosis surgery by using bilateral surgically-placed erector spinae plane catheters. Can J Anaesth 2020;67:621-622. 31776896 10.1007/s12630-019-01543-0

